# Combined Treatment With Dichloroacetic Acid and Pyruvate Reduces Hippocampal Neuronal Death After Transient Cerebral Ischemia

**DOI:** 10.3389/fneur.2018.00137

**Published:** 2018-03-12

**Authors:** Dae Ki Hong, A Ra Kho, Bo Young Choi, Song Hee Lee, Jeong Hyun Jeong, Sang Hwon Lee, Kyoung-Ha Park, Jae-Bong Park, Sang Won Suh

**Affiliations:** ^1^Department of Physiology, College of Medicine, Hallym University, Chuncheon, South Korea; ^2^Division of Cardiovascular Diseases, Hallym University Sacred Heart Hospital, Anyang, South Korea; ^3^Department of Biochemistry, College of Medicine, Hallym University, Chuncheon, South Korea

**Keywords:** transient cerebral ischemia, dichloroacetic acid, pyruvate, neuronal death, pyruvate dehydrogenase kinase

## Abstract

Transient cerebral ischemia (TCI) occurs when blood flow to the brain is ceased or dramatically reduced. TCI causes energy depletion and oxidative stress, which leads to neuronal death and cognitive impairment. Dichloroacetic acid (DCA) acts as an inhibitor of pyruvate dehydrogenase kinase (PDK). Additionally, DCA is known to increase mitochondrial pyruvate uptake and promotes glucose oxidation during glycolysis, thus enhancing pyruvate dehydrogenase (PDH) activity. In this study, we investigated whether the inhibition of PDK activity by DCA, which increases the rate of pyruvate conversion to adenosine triphosphate (ATP), prevents ischemia-induced neuronal death. We used a rat model of TCI, which was induced by common carotid artery occlusion and hypovolemia for 7 min while monitoring the electroencephalography for sustained isoelectric potential. Male Sprague-Dawley rats were given an intraperitoneal injection of DCA (100 mg/kg) with pyruvate (50 mg/kg) once per day for 2 days after insult. The vehicle, DCA only or pyruvate on rats was injected on the same schedule. Our study demonstrated that the combined administration of DCA with pyruvate significantly decreased neuronal death, oxidative stress, microglia activation when compared with DCA, or pyruvate injection alone. These findings suggest that the administration of DCA with pyruvate may enhance essential metabolic processes, which in turn promotes the regenerative capacity of the post-ischemic brain.

## Introduction

Transient cerebral ischemia (TCI) is the most commonly developed neurological disorder after heart attack or thromboembolism. When adequate blood circulation is restricted for only a short period of time, ischemic symptoms and sequelae are usually transient. However, if the cessation of blood circulation occurs for a prolonged period prior to the restoration of circulation, brain damage can be permanent. On the other hand, the process of blood recirculation following the cessation of blood flow may exacerbate certain aspects of brain injury, such as increased infarct volume and worsened clinical outcome after stroke. Thus, this phenomenon has been called “reperfusion injury” following ischemia (1).

During the reperfusion period after TCI, the blood supply restores glucose and oxygen levels that help to promote a return to physiological conditions. Nevertheless, excessive reactive oxygen species (ROS) are produced as a result and serve as primary drivers of the phenomenon of reperfusion injury, promoting pathological responses, such as leukocyte and proinflammatory neutrophil infiltration, complement and platelet activation, and the disruption of the blood–brain barrier (BBB), which are all components of this reperfusion injury. Therefore, reducing reperfusion injury is necessary for promoting resistance to cell death and regenerative capacity to repair ischemic tissues (1, 2). Previously, our lab suggested that not only the re-circulation of oxygen, but also the re-introduction of glucose was involved in this unwanted effect (3). We have demonstrated that re-infused glucose also induces cerebral reperfusion injury through nicotinamide adenine dinucleotide phosphate (NADPH) oxidase activation. Thus, we have termed this phenomenon “reperfusion injury” (3, 4). Either oxygen or glucose-induced reperfusion injury after ischemia is accompanied by multiple cellular and molecular responses, such as the activation of microglia (5) and BBB disruption (6, 7) as well as the production of ROS (8–10).

Dichloroacetic acid (DCA) has been used as an investigational therapeutic agent for multiple genetic mitochondrial diseases (11, 12). DCA has also been demonstrated to prevent mitochondrial apoptosis in cancer cells *in vivo* and *in vitro* (13, 14). DCA has been known as an inhibitor of mitochondrial enzyme pyruvate dehydrogenase kinase (PDK) that converts pyruvate to acetyl-coA, which is then oxidized in the mitochondria to produce energy in the citric acid cycle (15, 16). Therefore, DCA increases the flux of pyruvate into the mitochondria, therefore, enhancing glucose oxidation over the glycolytic pathway. The pyruvate dehydrogenase (PDH) enzyme complex (PDC) is localized to the mitochondrial inner membrane and promotes the reversible conversion of pyruvate to acetyl-CoA by decarboxylation, which is the rate-limiting step in the aerobic oxidation of glucose, pyruvate, and lactate. These enzymes promote this by modifying its phosphorylation and protein stability (17, 18). DCA has been demonstrated to increase the uptake of pyruvate into the mitochondria and improve the ratio of glucose oxidation during the glycolysis process, thus enhancing PDH activity (19, 20). Based on these chemical characteristics, we investigated whether the inhibition of PDK activity by DCA, which increases the rate of pyruvate converted to adenosine triphosphate (ATP), prevents neuronal damage after TCI. In this study, we found that DCA treatment with pyruvate reduced the number of Fluoro-Jade B (FJB) positive neurons after TCI when compared to the vehicle-treated group.

Pyruvate can be produced from glucose through glycolysis and contributes energy to cells *via* the tricarboxylic acid (TCA) cycle when oxygen is present (21, 22). It has been shown that neuroprotective effects can be observed following treatment with pyruvate in various neurological diseases, such as TCI (23, 24), traumatic brain injury (25, 26), and hypoglycemia (27, 28). Pyruvate administration showed transient benefits, such as reduced infarct volume and increased ATP levels in the brain after middle cerebral artery occlusion (29). In addition, pyruvate treatment reduces ischemic neuronal death and promotes the survival of hippocampal neurons (23). After traumatic brain injury, pyruvate treatment decreases degenerating neurons in the hippocampal CA3 region and microglia activation in the ipsilateral temporal cortex (25). Our lab has also demonstrated that pyruvate administration reduced hypoglycemia-induced neuronal death (27, 30).

Following the logic that pyruvate has clear neuroprotective effects on various neurological diseases, we hypothesized that DCA could attenuate TCI-induced neuronal death by increasing the ratio of acetyl-coA from pyruvate for energy generation and increasing glucose oxidation and utilization. Although a previous study demonstrated that a dose of DCA (225 mg/kg) led to neuroprotective effects after TCI in gerbil, no further evaluation has been performed using different ischemia models or animals (31). In this study, we hypothesized that the combined administration of a low dose of DCA (100 mg/kg) with a low dose of pyruvate (50 mg/kg) may be an ideal therapeutic tool for preventing TCI-induced neuronal death. Here, we demonstrate for the first time that intraperitoneal administration of DCA with pyruvate showed a significant reduction in neuronal death after TCI.

## Materials and Methods

### Ethics Statement

Animal studies and approved experimental procedures were in strict compliance with the guidelines of the Institutional Animal Studies Care and Use Committee of the Hallym University (Protocol # Hallym-2016-67). Animals were sacrificed using isoflurane anesthesia and experimental procedures minimized animal suffering.

### Experimental Animals

Eight-week-old Sprague-Dawley male rats were used in this study (250–350 g, DBL Co, Chungcheongbuk, Korea). They were housed in a state of consistently maintained temperature and humidity (22 ± 2°C, 55 ± 5%, and 12-h light/dark cycle) with food (Purina, Gyeonggi, Korea), and water *ad libitum*. This manuscript was written up in accordance with the ARRIVE (Animal Research: Reporting *in Vivo* Experiments) guidelines (32).

### TCI Induction

Bilateral common carotid artery occlusion and hypotension is a well-known TCI induction method (33). Rats were anesthetized using 3% isoflurane and ventilated with 30% oxygen and 70% nitrous oxide (Air Liquid America, Houston, TX, USA). A catheter filled with heparin was cannulated in the femoral artery for draining blood and monitoring blood pressure. In the temporal area of the skull, bilateral burr holes were made to place the electroencephalography probes. Next, two monopolar electrodes were located under the dura mater and another reference electrode was placed in the neck muscle. During the ischemic surgery, the experimental animal body temperature was maintained at 36.5–37.5°C using a heating pad (3, 34). Systemic mean arterial pressure was decreased 40–45 mmHg by draining blood (7–10 mL) from the femoral artery using a heparinized syringe. When the arterial pressure decreased, the bilateral common carotid arteries were clamped. Proper induction of TCI was verified by the existence of isoelectricity on the electroencephalography (3, 35). After observation, isoelectric electroencephalography signal was taken and 7 min later, blood perfusion was reinstated by unclamping the bilateral common carotid arteries and blood flowed back through the femoral artery. Recovery after reperfusion was verified by a return to the baseline electroencephalography signal. DCA in 1.5 mL of 0.9% normal saline were immediately administrated to the DCA treatment group through the intraperitoneal space. The vehicle control group was only administrated with 0.9% normal saline. In addition, sham-operated groups were administrated on the same schedule with 0.9% normal saline. After surgery, animals were closely observed for 3 h in an incubator consistently maintained at 36°C. After observation, they were moved to the temperature and humidity controlled recovery room.

### DCA and Pyruvate Administration

To evaluate the effect of DCA with pyruvate on TCI-induced neuronal death, the experimental groups were divided into eight groups [control (vehicle, DCA, pyruvate, and DCA with pyruvate) and TCI (vehicle, DCA, pyruvate, and DCA with pyruvate)]. The DCA and pyruvate-treated groups were administrated DCA (100 mg/kg, i.p.) and pyruvate (50 mg/kg, i.p.) dissolute in normal saline. TCI operated animals were administrated DCA with pyruvate once per day for 2 days. First, after TCI induction, we immediately injected the drugs into the intraperitoneal space. Next day, we injected once more. The vehicle group was injected on the same schedule with 0.9% normal saline. All groups were sacrificed at 1 week following TCI.

### Brain Sample Preparation for Immunostaining

To investigate the neuroprotective effects of DCA with pyruvate, experimental animals were sacrificed after 1 week following TCI. They were deeply anesthetized with urethane (1.5 g/kg, i.p.) and transcardially perfused with 0.9% saline, followed by 4% paraformaldehyde. Next, the brains were extracted and post-fixed for approximately 1 h in 4% paraformaldehyde. After post-fixation, the brain was immersed in 30% sucrose solution for ~2–3 days for cryoprotection. Later, after the brain samples sank to the bottom of tube, brain samples were frozen on the freezing medium and sliced in the cryostat at 30 µm thicknesses. Brain slices were stored in a storage solution until used for immunohistochemistry and immunofluorescence staining.

### Detection of Degenerating Neurons

To evaluate neuronal death after TCI, brain sections (30 µM) were put on gelatin-coated slides (Fisher Scientific, Pittsburgh, PA, USA). To detect degenerating neurons, brain sections were stained by the FJB staining method (36, 37). Brain sections were soaked in ethanol in the order of 100, 70%, and distilled water. Then, sections were deeply immersed in 0.06% potassium permanganate solution for 15 min. After 15 min, we rinsed them in distilled water for 1 min three times. Then, they were reacted in 0.001% FJB (Histochem Inc., Jefferson AR) for 30 min and rinsed for 10 min three times in distilled water. After rinsing, slides were dried by gentle air flow (Labtech, Co.), dehydrated in xylene, and mounted with DPX (Sigma-Aldrich Co., St. Louis, MO, USA). To verify TCI-induced neuronal death, it was observed with a fluorescence microscope using blue (450–490 m) wavelength. We used about 6–8 coronal brain sections that were gathered from each animal by starting 4.0 mm posterior from the bregma. To correctly count the FJB-positive cells, we used a blind observer. They counted the FJB-positive cells in the hippocampal subiculum, cornus ammonis 1 (CA1), and CA2 of the bilateral hemisphere. The total number of FJB-positive cells from each hippocampal region were used for statistical analysis.

### Detection of Neuronal Survival

To evaluate the survival of hippocampal neurons after TCI, brain sections were stained by NeuN staining. Tissues were reacted with monoclonal anti-mouse-NeuN antiserum (diluted 1:500, Billerica, Millipore Co., Boston, MA, USA) in phosphate buffered saline (PBS) consisting of 0.3% Triton X-100 overnight in an incubator at 4°C. After incubation, brain sections were rinsed three times for 10 min with PBS. Then, these sections were incubated with biotinylated anti-mouse IgG serum (diluted 1:250, Vector, Burlingame, CA, USA), followed by the Avidin-Biotin Complex (ABC) mixture (Vector, Burlingame, CA, USA) for 2 h at room temperature. The immunoreactivity was visualized with 0.06% 3,3’-diaminobenzidine (DAB ager, Sigma-Aldrich Co., St. Louis, MO, USA) in 0.1 M PBS buffer. NeuN (+) neurons were observed using a bright-field microscope (Olympus, Japan). The captured image was counted by a blind observer. NeuN (+) neurons were counted in the hippocampal subiculum, CA1, and CA2 of the bilateral hemisphere. Then, the total counted number of NeuN (+) neurons was divided by two and averaged from five sections and then used for statistical analysis.

### Detection of Oxidative Injury

To detect oxidative injury induced by the lipid peroxidation product from brain sections, 4-hydroxy-2-noneal (4HNE) was conducted by immunofluorescence staining. 4HNE antibodies (Alpha Diagnostic Intl. Inc., San Antonio, TX, USA) for immunohistochemical staining have been explained in our previous study (4, 38). In brief, brain sections were incubated in a mixture of polyclonal rabbit anti-HNE antiserum (diluted 1:500, Alpha Diagnostic Intl. Inc., San Antonio, TX, USA) in PBS consisting of 0.3% Triton X-100 overnight in an incubator maintained at 4°C. After primary antibody incubation, they were rinsed three times for 10 min with PBS. After washing, brain sections were incubated in a mixture of Alexa Fluor 594-conjugated goat anti-rabbit IgG secondary antibody (diluted 1:250, Invitrogen, Grand Island, NY, USA) for 2 h at room temperature. These sections were photographed with a fluorescence microscope and the intensity of the 4HNE fluorescence was evaluated using ImageJ (NIH, Bethesda, MA, USA).

### Detection of BBB Disruption

To estimate the putative breakdown of the BBB, we conducted immunohistochemistry to find serum IgG leakage (39). The ABC immunoperoxidase protocol was used to detect IgG-like immunoreactivity (40). Rat brains were fixed by cardiac perfusion with 0.9% normal saline and then followed by 4% paraformaldehyde. We used anti-rat IgG (diluted 1:250, Burlingame, Vector, CA) that can detect leakages of IgG when the BBB is disrupted by external and internal inserts. After washing in PBS, tissues were deeply immersed in the ABC complex mixture (Vector, Burlingame, CA, USA) for 2 h at room temperature on the shaker gently. The immunoreactivity was visualized with 0.06% 3,3’-diaminobenzidine (DAB ager, Sigma-Aldrich Co., St. Louis, MO, USA) in 0.1 M PBS buffer. Revealed IgG extravasations were observed using the bright-field microscope.

### Evaluation of Microglial Activation

To elucidate the potential protective effects of DCA with pyruvate, we investigated the degree of microglial activation using the conjugated donkey anti-mouse (CD11b) (diluted 1:500, AbD Serotec, UK) and Iba1 (diluted 1:500, ab5076, Abcam, UK) antibodies. About 6–8 sections from each animal were investigated for microglial activation. After washing in 1 mM PBS, immunostaining was conducted with a mouse anti-rat containing blocking buffer (10% goat serum and 0.1% Triton X-100 in 1 mM PBS) overnight in the incubator maintained at 4°C. After rinsing, the sections were immersed in a mixture of Alexa Fluor 488-CD11b/goat (Iba1) IgG secondary antibody (diluted 1:250, Molecular Probes, Invitrogen) for 2 h at room temperature, gently on the shaker. These sections were photographed with fluorescence microscope and intensity of CD11b and Iba1 fluorescence was measured using ImageJ program (NIH, Bethesda, MA, USA).

### Statistical Analysis

Numerical data were displayed as the mean ± SEM. Analysis of variance according to the Bonferroni *post hoc* test was used to compare each experimental group. Statistical significance was described as *P* < 0.05.

## Results

### DCA With Pyruvate Decreases TCI-Induced Neuronal Death

**Figure 1:**
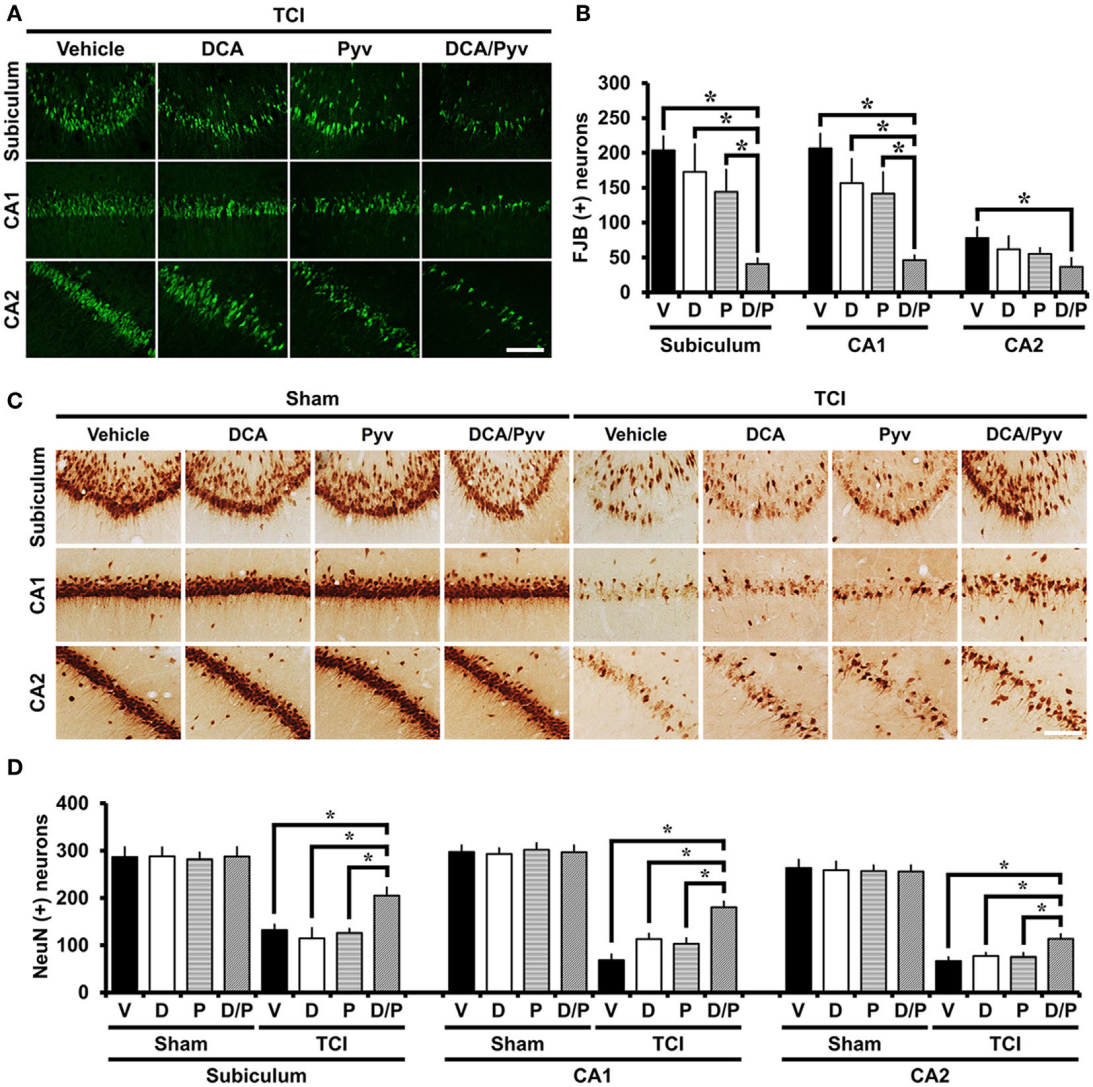
Combined treatment with dichloroacetic acid (DCA) and pyruvate reduces transient cerebral ischemia (TCI)-induced neuronal death. This figure shows that the combined treatment of DCA and pyruvate demonstrated neuroprotective effects after TCI. Brain sections were stained by Fluoro-Jade B (FJB) to analyze the neurodegeneration. **(A)** FJB positive neurons were observed in the subiculum (Subi), cornus ammonis 1 (CA1), and CA2. Co-treatment of DCA with pyruvate once per day for 2 days inhibited neuronal death in the subiculum, CA1, and CA2 after TCI. There was no significant difference in DCA-only or pyruvate-only treatment when compared to the vehicle-treated group after TCI. Scale bar = 100 µm. **(B)** Bar graph represents the quantified degenerating neurons. The statistically significant difference is indicated between the DCA + pyruvate-treated groups and the vehicle-treated groups. Administration of DCA with pyruvate reduced the number of FJB (+) neurons in the subiculum, CA1, and CA2 areas when compared to the vehicle-treated group (TCI-vehicle, *n* = 11; TCI-DCA, *n* = 6; TCI-pyruvate, *n* = 7; TCI-DCA + pyruvate, *n* = 8). **(C)** Survival of hippocampal neurons were evaluated by NeuN staining. There was a significant difference between the TCI-vehicle, TCI-DCA, and TCI-pyruvate to the TCI-DCA + pyruvate-treated groups. The combined treatment of DCA with pyruvate improved neuronal survival in the hippocampal subiculum, CA1, and CA2 regions after TCI. Scale bar = 100 µm. **(D)** Bar graph represents the quantified NeuN (+) neurons. Data show that there was a statistically significant difference between the TCI control groups and the combined treatment group (DCA with pyruvate) after TCI groups (TCI-vehicle, *n* = 5; TCI-DCA, *n* = 5; TCI-pyruvate, *n* = 5; TCI-DCA + pyruvate, *n* = 5). Data are mean ± SEM. *Significant difference from the vehicle-treated group. *P* < 0.05. D, DCA; P, pyruvate; D/P, DCA + pyruvate; V, vehicle.

To estimate whether the combined treatment of DCA and pyruvate had a neuroprotective effect after TCI-induced neuronal death, rats received treatment with or without these agents once per day for 2 days after TCI. At 7 days after TCI, degenerating neurons were evaluated in the hippocampal areas. Degenerating neurons were visualized by using the fluorescent dye FJB, which has a high specificity for degenerating apoptotic and necrotic neurons (41). When compared with the control groups, rats given DCA with pyruvate after TCI showed dramatically reduced hippocampal neuronal death in the subiculum, CA1, and cornus ammonis 2 (CA2). Although there was a slight decrease in the number of degenerating neurons after TCI, the sole administration of a low dose of DCA (100 mg/kg) or pyruvate (50 mg/kg) showed no significant reduction of degenerating neurons after TCI (Figure 1A). Figure 1B represents the number of detected FJB-positive neurons from the hippocampal areas. The combined administration of DCA with pyruvate showed an approximately 79% reduction in the number of FJB (+) neurons in the subiculum (TCI-vehicle, 203.5 ± 20.1; TCI-DCA + pyruvate, 40.6 ± 7.8), 76% in the CA1 (TCI-vehicle, 206.4 ± 20.8; TCI-DCA + pyruvate, 46.1 ± 6.8), and 53% in the CA2 (TCI-vehicle, 77.9 ± 15.1; TCI-DCA + pyruvate, 36.6 ± 12.3) when compared to the control group (Figure 1B).

### DCA With Pyruvate Improves Survival of Hippocampal Neurons After TCI

Following TCI, many neurons degenerate and disappear. To investigate whether the combined treatment of DCA and pyruvate promoted neuronal survival after TCI, we performed NeuN staining, a specific marker for neuronal nuclei (42). After TCI, brain sections showed obvious neuronal loss across the entire hippocampus. Compared with the sham groups, most NeuN (+) neurons were depleted after TCI in subiculum, CA1, and CA2 regions. However, in the case of the combined treatment of DCA with pyruvate after TCI, a significant number of NeuN (+) neurons survived in the hippocampal regions when compared to the vehicle, DCA-only, or pyruvate-only administrated groups after TCI. These results demonstrated that the combined treatment of DCA with pyruvate showed neuroprotective effects even several days after TCI (Figure 1C). Figure 1D represents the quantified NeuN (+) neurons in each hippocampal region. Compared with the vehicle-treated group, the combined treatment of DCA with pyruvate showed a greater number of NeuN (+) neurons; about 64% in the subiculum (TCI-vehicle, 132.1 ± 11.4; TCI-DCA + pyruvate, 205.2 ± 17.1), 163% in the CA1 (TCI-vehicle, 68.4 ± 12.4; TCI-DCA + pyruvate, 180.6 ± 12.1), and 68% in the CA2 (TCI-vehicle, 67.1 ± 7.8; TCI-DCA + pyruvate, 113.5 ± 9.6).

### DCA With Pyruvate Reduces TCI-Induced Oxidative Injury

**Figure 2:**
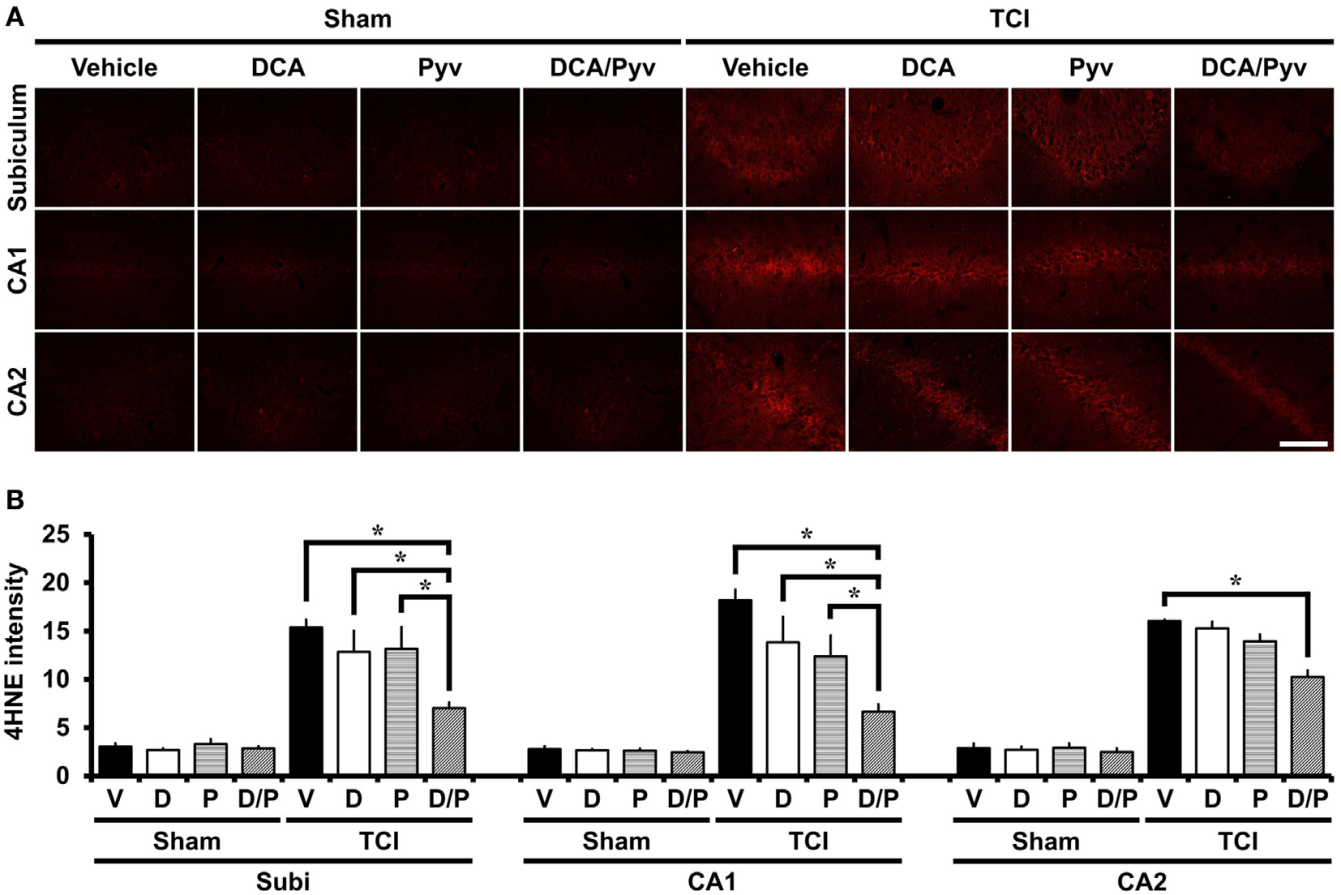
Combined treatment with dichloroacetic acid (DCA) and pyruvate reduces oxidative injury after transient cerebral ischemia (TCI). Neuronal oxidative injury was measured by 4-Hydroxy-Nonenal (4HNE, red color) staining at the hippocampal cornus ammonis 1 (CA1), CA2, and subiculum areas at 7 days after TCI. **(A)** Sham-operated groups showed minimal 4HNE immunoreactive signal across the hippocampal regions. Combined treatment of DCA with pyruvate reduced the immunoreactive fluorescence intensity of 4HNE in the hippocampus when compared to the vehicle-treated group after TCI. Scale bar = 100 µm. **(B)** Bar graph represents statistically significant difference between groups (Normal-vehicle, *n* = 5; Normal-DCA, *n* = 5; Normal-pyruvate, *n* = 5; Normal-DCA + pyruvate, *n* = 5; TCI-vehicle, *n* = 6; TCI-DCA, *n* = 4; TCI-pyruvate, *n* = 4; TCI-DCA + pyruvate, *n* = 4). Data are mean ± SEM. *Significant difference from the vehicle-treated group, *P* < 0.05. D, DCA; P, pyruvate; D/P, DCA + pyruvate; V, vehicle.

Immediately after TCI, ROS have been observed to be produced in many brain areas, including the hippocampus and cerebral cortex, playing important roles in several cell death pathways, including neuronal plasma membrane damage and mitochondrial dysfunction after ischemia (43). To test whether ROS production and lipid peroxidation is induced in hippocampal neurons after TCI, we performed 4HNE staining, which is often used as a specific marker for visualizing oxidative stress after various brain diseases (44). The sham-operated groups, including normal saline, DCA, or the pyruvate-injected groups showed no difference with respect to the 4HNE fluorescence signal in the hippocampal regions. 7 days after TCI increased, 4HNE fluorescence intensities in the hippocampal areas were observed. However, the combined treatment of DCA with pyruvate showed a significant reduction of 4HNE intensities when compared to the control groups (Figure 2A). Figure 2B represents the analyzed intensity of the 4HNE fluorescence signal from each of the hippocampal regions. Oxidative stress was reduced by 63% in the DCA with pyruvate co-administered group after TCI, when compared with the control groups in CA1 (TCI-vehicle, 18.19 ± 1.07; TCI-DCA + pyruvate, 6.66 ± 0.73), 61% in CA2 (TCI-vehicle, 16.05 ± 0.14; TCI-DCA + pyruvate, 10.26 ± 0.65), and 54% in the subiculum (TCI-vehicle, 15.38 ± 0.79; TCI-DCA + pyruvate, 7.01 ± 0.59) (Figure 2B).

### DCA With Pyruvate Has a Protective Effect on TCI-Induced BBB Disruption

**Figure 3:**
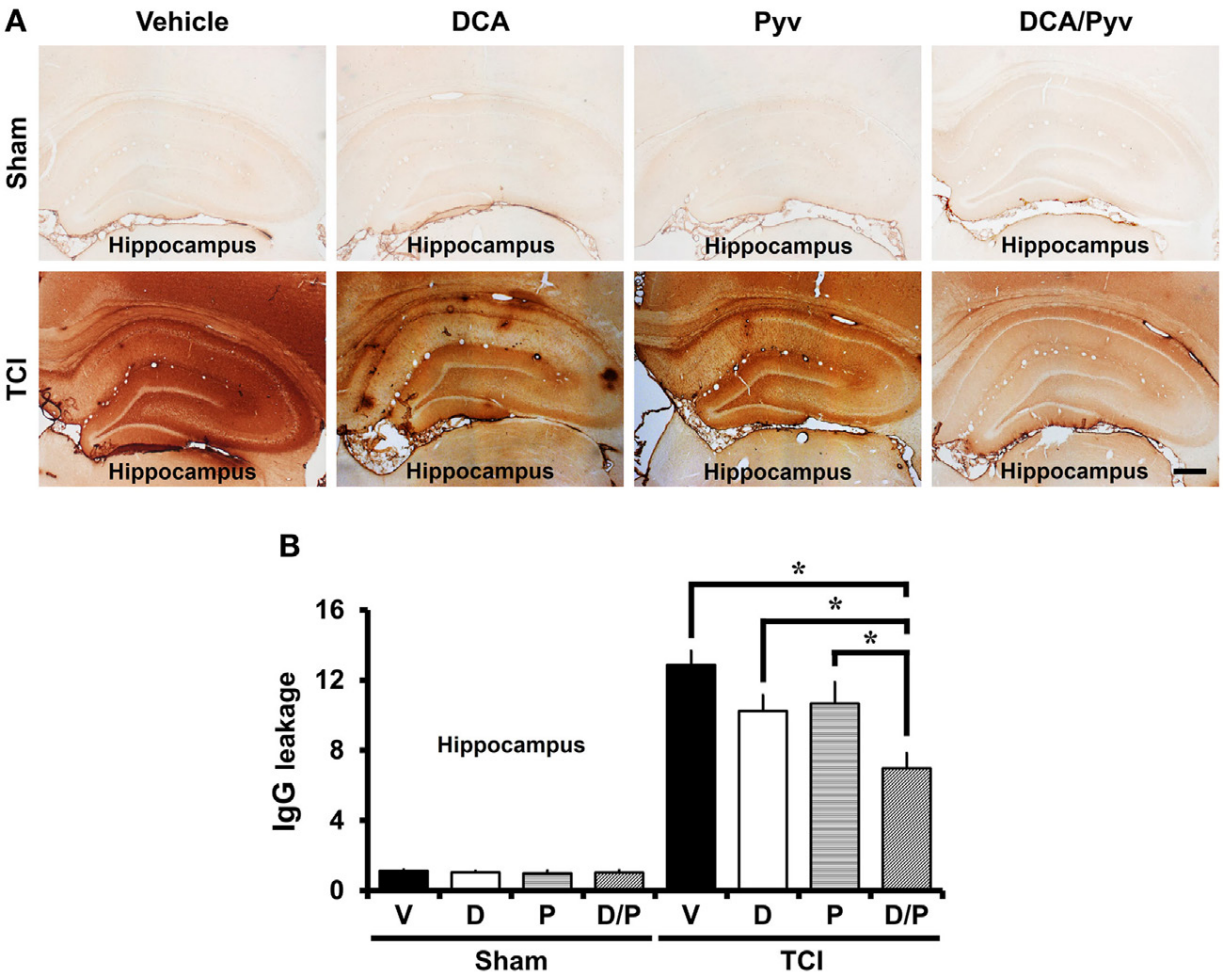
Combined treatment with dichloroacetic acid (DCA) and pyruvate reduces blood–brain barrier (BBB) disruption after transient cerebral ischemia (TCI). TCI-induced BBB disruption is reduced by the combined treatment of DCA and pyruvate. **(A)** shows low magnification (4×) of a microscopic image of IgG staining in the while hippocampal regions in each group. These images show that BBB disruption occurred after TCI. Combined treatment of DCA and pyruvate after TCI reduced the leakage of serum IgG in the whole hippocampus when compared to the vehicle-treated group. Scale bar = 500 µm. **(B)** Bar graph represents the quantification of IgG serum extravasation in the whole hippocampus (Normal-vehicle, *n* = 3; Normal-DCA, *n* = 3; Normal-pyruvate, *n* = 3; Normal-DCA + pyruvate, *n* = 3; TCI-vehicle, *n* = 8; TCI-DCA, *n* = 5; TCI-pyruvate, *n* = 6; TCI-DCA + pyruvate, *n* = 11). Data are mean ± SEM. *Significant difference from the vehicle-treated group. *P* < 0.05. D, DCA; P, pyruvate; D/P, DCA + pyruvate; V, vehicle.

Transient cerebral ischemia is accompanied by BBB disruption and vascular permeability changes. These changes exacerbate neuronal death and cause BBB disruption, followed by leakage of serum IgGs into the brain (6). To examine BBB disruption, we performed IgG staining that was visualized by extravasation of serum IgG by immunohistochemistry (39). Endogenous IgGs were restricted to vessels in non-insulted sham animals. No difference was observed when comparing Sham-vehicle-treated animals to Sham-DCA with pyruvate-treated animals. In comparison, in TCI-induced rats, we found extravasation of serum IgG throughout the entire hippocampus (Figure 3A). Figure 3B shows a graph of the extent of IgG extravasation serum from the disrupted BBB in the various hippocampal regions. The leakage of IgGs decreased by 36% in the DCA with pyruvate group after ischemic insult when compared with the TCI control group in all hippocampal regions (TCI-vehicle, 12.85 ± 0.81; TCI-DCA + pyruvate, 6.96 ± 0.38) (Figure 3B).

### DCA With Pyruvate Decreases TCI-Induced Microglial Activation

**Figure 4:**
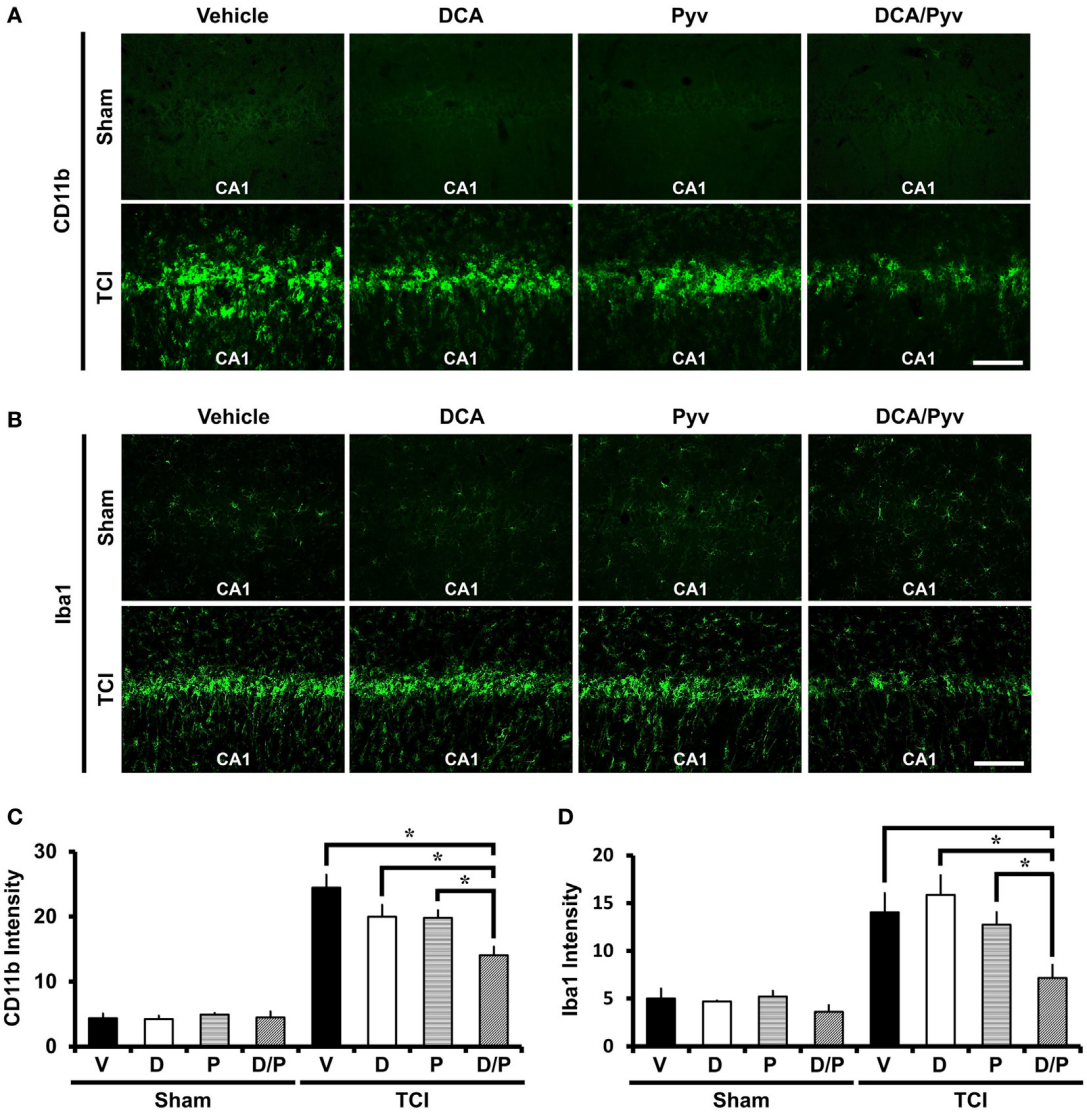
Combined treatment with dichloroacetic acid (DCA) and pyruvate reduces transient cerebral ischemia (TCI)-induced microglia activation. TCI-induced microglial activation was reduced by the co-treatment of DCA and pyruvate. **(A)** Conjugated donkey anti-mouse (CD11b) fluorescence microscopic images in the cornus ammonis 1 (CA1) region, which shows microglia and macrophage activation in the TCI-induced groups. CD11b expressed fluorescence signal was reduced in the combined treatment of the DCA and pyruvate group, compared to the vehicle-treated group. Scale bar = 100 μm (Normal-vehicle, *n* = 5; Normal-DCA, *n* = 5; Normal-pyruvate, *n* = 5; Normal-DCA + pyruvate, *n* = 5; TCI-vehicle, *n* = 5; TCI-DCA, *n* = 5; TCI-pyruvate, *n* = 7; TCI-DCA + pyruvate, *n* = 7). **(B)** Iba1 fluorescence microscopic images in the hippocampal CA1, which shows specific expression in microglia within the brain (Normal-vehicle, *n* = 5; Normal-DCA, *n* = 5; Normal-pyruvate, *n* = 5; Normal-DCA + pyruvate, *n* = 5; TCI-vehicle, *n* = 5; TCI-DCA, *n* = 5; TCI-pyruvate, *n* = 6; TCI-DCA + pyruvate, *n* = 5). **(C,D)** The bar graph represents the intensity of activated CD11b and Iba1 in the CA1 area that was based on CD11b and Iba1 fluorescence signal. Data are mean ± SEM, *n* = 5–7 from each group. D, DCA; P, pyruvate; D/P, DCA + pyruvate; V, vehicle.

Ischemic insult induces several pathological changes, such as an enhanced neuroinflammatory response that leads to the activation and proliferation of microglia. These migrate to the region of ischemic insult and are able to change their activity states depending on around the environmental statement (45, 46). During brain injury, they rapidly change their endogenous program by transforming morphology, proliferating the release of chemokines, cytokines, and the other inflammatory compounds (5, 47). To evaluate the neuroinflammatory response after TCI, we conducted CD11b and Iba1 immunostaining. CD11b is well known as marker for microglia and macrophages. In the brain, Iba1 is specifically expressed in the microglia. Compared with the sham- and vehicle-treated groups, Sham-DCA with the pyruvate-treated groups showed no difference in microglia activation intensity signal. After TCI, CD11b and Iba1 fluorescence signals remarkably increased in intensity. However, combined treatment of DCA with pyruvate reduced CD11b activation (Figure 4A) and Iba1 activation (Figure 4B). Figures 4C,D represents the analyzed intensity of the CD11b and Iba1 fluorescence signals in the hippocampal CA1 region. These fluorescence signals were reduced by about 43% (CD11b), 49% (Iba1) in the combined treatment of the DCA and pyruvate groups after TCI when compared to TCI-vehicle-treated groups (TCI-vehicle, 24.45 ± 1.96; TCI-DCA + pyruvate, 14.03 ± 1.30) (Figure 4C), (TCI-vehicle, 14.02 ± 2.01; TCI-DCA + pyruvate, 7.16 ± 1.37) (Figure 4D).

## Discussion

**Figure 5:**
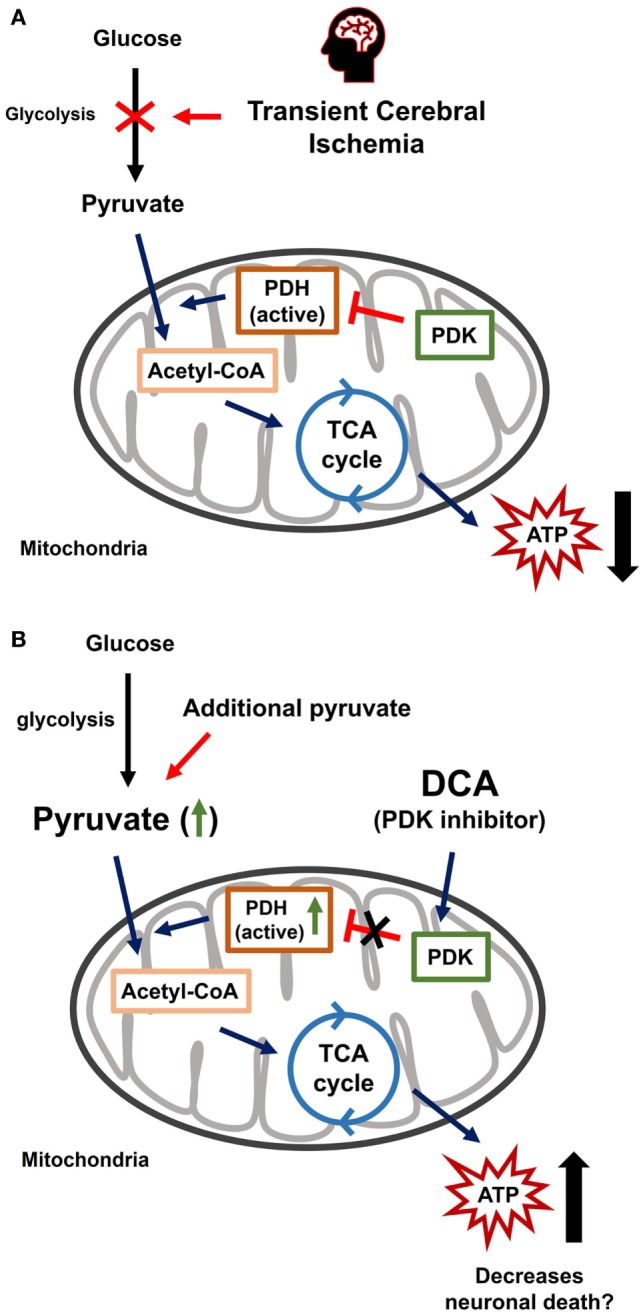
Transient cerebral ischemia (TCI)-induced neuronal death and effects of combined dichloroacetic acid (DCA) with pyruvate. **(A)** The TCI-induced neuronal death mechanism is diverse and well known. One of these mechanisms begins by blocking glycolysis at the step that converts glucose into pyruvate for energy production. It is upstream of the process that sequesters pyruvate and transports it to the mitochondria and tricarboxylic acid (TCA) cycle. Reducing pyruvate production can be caused by TCI. The pyruvate decline means that the amount of acetyl-coenzyme-A entering the TCA cycle will decrease. As a result, the production of adenosine triphosphate decreases and contributes to TCI-induced hippocampal neuronal death. **(B)** Combined DCA with pyruvate administration increases energy production capacity. Reduced pyruvate supply due to TCI can inhibit neuronal death due to the additional administration of pyruvate. Pyruvate dehydrogenase kinase, an inhibitor of DCA is administrated to make energy production more efficient.

This study investigated whether the combined treatment of DCA and pyruvate have neuroprotective effects in the hippocampus after TCI. A low dose of DCA or pyruvate alone has shown no beneficial effects on TCI-induced neuronal death. However, the combined administration of DCA (PDK inhibitor) with pyruvate showed significant neuroprotective effects. These results suggested that the combined treatment of DCA with pyruvate might be used as a therapeutic agent after stroke (Figure 5).

Transient cerebral ischemia results from a lack of oxygen and metabolite supply to the central nervous system. Due to this lack of blood flow, mitochondria begin to dysfunction, leading to the production of ROS and other neuronal death processes, which are initiated almost immediately after TCI. DCA has been known to be an inhibitor of PDK, which increases the uptake of pyruvate into the mitochondria, and promotes glucose oxidation during glycolysis. Pyruvate is a major metabolite from glucose, which is used to support further energy processes. A previous study has demonstrated that DCA showed a protective effect in neurological metabolic diseases, such as cerebral ischemia (31). In addition, our previous study demonstrated that hypoglycemic insult-induced brain damage was also reduced by pyruvate administration. The aim of this study was to identify whether DCA showed neuroprotective effects after TCI if combined with pyruvate.

Many mechanisms that are responsible for neuronal death after TCI have been elucidated. For example, calcium overload after glutamate binds to N-methyl-d-aspartate and α-amino-3-hydroxy-5-methyl-4-isoxazolepropionic acid receptors cause degradation of essential proteins and membranes (48). After TCI, ROS are generated, directly damaging lipids, proteins, and nucleic acids (49), and poly ADP-ribose polymerase is also activated (50). However, several studies have suggested that NADPH oxidase is also a major source of neuronal ROS production in hypoglycemia and other conditions (4, 51). All of these scenarios can lead to neuronal cell death. Our lab has previously searched for agents that have positive effects, such as reducing neuronal death, improving survival of neurons, and decreasing microglial activation.

Dichloroacetic acid is well known as a PDK inhibitor in mitochondria. This agent enhances the conversion of acetyl-coenzyme-A (acetyl-CoA) from pyruvate, and improves PDH activity, which controls pyruvate entry into the TCA cycle. Under the ischemic state, a lack of oxygen leads to dysfunction in ATP production, leading to energy failure in neurons and a collapse of ATP-dependent ion transport pumps. A previous study on DCA for anti-cancer agents that targeted metabolism has been reported (15, 52).

In previous studies, researchers found that DCA treatment increased glucose oxidation, which includes glycolysis, the TCA cycle, electron transport system, and lactate oxidation in neurons and astroglia (19). Furthermore, DCA has a potential use in targeted metabolic therapy (15). In addition, our lab demonstrated that pyruvate has neuroprotective effects and reduced cognitive impairment after severe hypoglycemia (27).

Thus, we speculate that ischemic brain damage blocks the step between glucose and GAPDH in the glycolysis pathway, particularly glucose-6-phosphate, which can be converted to ribose-5-phosphate, concurrently producing NADPH. The NADPH can be utilized as a substrate for NADPH oxidase to produce superoxide. Thereby, supplying pyruvate instead of glucose can, which can be used as energy for ATP production through avoiding ischemic action to inhibit GAPDH. Here, PDH activation by DCA is required for pyruvate oxidation.

To determine whether DCA and pyruvate had a neuroprotective effect, we conducted several immunohistochemistry and immunofluorescence staining protocols. First, we detected degenerating neurons by FJB staining (36). In this study, unfortunately, we found that only DCA or pyruvate administration once per day for 2 days after TCI had no effect when compared with the TCI-vehicle-treated group. Therefore, we changed our hypothesis that either DCA or pyruvate should have neuroprotective effects alone after TCI to test the combined treatment of DCA with pyruvate. Consequently, the combined DCA with pyruvate treatment remarkably reduced hippocampal neuronal death after TCI, as observed by FJB staining. Subsequently, additionally we detected the survival of hippocampal neurons using NeuN staining. This process confirmed how many decreased degenerating neurons after TCI survived after the combined DCA with pyruvate treatment. Successfully, we found that the combined DCA with pyruvate administration decreased hippocampal neuronal death as well as improved the survival of hippocampal neurons after TCI in the hippocampal subiculum, CA1, and CA2 regions.

Reactive oxygen species production stimulated by ischemic injury due to excessive mitochondrial oxygen radical generation (53) and free radicals are also produced during the inflammatory response after TCI. In addition to the production of oxidants, the deactivation of detoxification systems and scavenging antioxidants are also included in injurious brain ischemia processes (54). To determine whether the neuroprotective effect of the combined DCA with pyruvate was associated with antioxidant effects in hippocampal regions, we conducted 4HNE staining to detect oxidative stress. As a result of 4HNE staining, we found that the combined DCA with pyruvate had neuroprotective effects, and also reduced ROS production in the hippocampal subiculum, CA1, and CA2 regions when compared to the TCI-vehicle-treated group.

The brain damage caused by TCI, hypoglycemia, traumatic brain injury, and epilepsy produce neuroinflammatory responses. Microglia is the macrophage of the central nervous system. Downstream of TCI, inflammatory cytokines, and TNF-α, IL-1β are produced, and activated microglia (involving astrocytes) migrates to the injured site (45). Additional research demonstrated that after 48 h following middle cerebral artery occlusion, the peak of microglial and macrophage infiltration was reached (55, 56). These findings imply that microglia are components of brain injury and play an essential role in TCI. To evaluate neuroinflammatory responses after TCI in the given combined DCA with the pyruvate group and vehicle-treated group, we conducted CD11b and Iba1 staining. Remarkably, the combined DCA with pyruvate inhibited CD11b and Iba1 expression after TCI.

Taken together, this study suggests that the combined DCA and pyruvate treatment has a role as a potential therapeutic agent for reducing TCI-induced hippocampal neuronal death. However, since the combined DCA with pyruvate was administrated immediately after insult, further research is needed to discover more clinically actionable time windows for treatment.

## Conclusion

This study supports the hypothesis that administration of the combined treatment of DCA with pyruvate prevents neuronal death in the hippocampus after ischemia. This study suggests that these agents have a role in potential therapeutic application after TCI and may serve as a beneficial treatment for the improvement of cognitive function in ischemic patients.

## Ethics Statement

Animal studies and approved experimental procedures were in strict compliance with the guidelines of the Institutional Animal Studies Care and Use Committee of the Hallym University (Protocol # Hallym-2016-67).

## Author Contributions

DH researched the data, reviewed, and edited the manuscript. Song Hee L, AK, JJ, and Sang Hwon L researched the data. BC reviewed and edited the manuscript. KP and JP researched the data, reviewed, edited, and wrote the manuscript. SS contributed to the discussion, reviewed, edited, and wrote the manuscript.

## Conflict of Interest Statement

None of the authors have any financial conflicts of interest relevant to this work.
